# Investigating the Association between Nutrient Intake and Food Insecurity among Children and Adolescents in Palestine Using Machine Learning Techniques

**DOI:** 10.3390/children11060625

**Published:** 2024-05-23

**Authors:** Radwan Qasrawi, Sabri Sgahir, Maysaa Nemer, Mousa Halaikah, Manal Badrasawi, Malak Amro, Stephanny Vicuna Polo, Diala Abu Al-Halawa, Doa’a Mujahed, Lara Nasreddine, Ibrahim Elmadfa, Siham Atari, Ayoub Al-Jawaldeh

**Affiliations:** 1 Department of Computer Sciences, Al-Quds University, Jerusalem P.O. Box 20002, Palestine; 2 Department of Computer Engineering, Istinye University, Istanbul 34010, Turkey; 3 Department of Nutrition and Food Technology, College of Agriculture, Hebron University, Hebron P.O. Box 40, Palestine; 4 Institute of Community and Public Health, Birzeit University, Ramallah P.O. Box 14, Palestine; 5 Nutrition Department, Ministry of Health, Ramallah P.O. Box 4284, Palestine; 6 Nutrition and Food Technology Department, Faculty of Agriculture and Veterinary Medicine, An-Najah National University, Nablus P.O. Box 7, Palestine; 7 Faculty of Medicine, Al-Quds University, Jerusalem P.O. Box 20002, Palestine; 8 Nutrition and Food Sciences Department, Faculty of Agriculture and Food Sciences, American University of Beirut, Beirut 1107 2020, Lebanon; 9 Department of Nutrition, Faculty of Life Sciences, University of Vienna, 1090 Vienna, Austria; 10 Regional Office for the Eastern Mediterranean, World Health Organization, Cairo 7608, Egypt

**Keywords:** food insecurity, nutrient intake, machine learning, sociodemographic factors, socioeconomic disparities, children

## Abstract

Food insecurity is a public health concern that affects children worldwide, yet it represents a particular burden for low- and middle-income countries. This study aims to utilize machine learning to identify the associations between food insecurity and nutrient intake among children aged 5 to 18 years. The study’s sample encompassed 1040 participants selected from a 2022 food insecurity household conducted in the West Bank, Palestine. The results indicated that food insecurity was significantly associated with dietary nutrient intake and sociodemographic factors, such as age, gender, income, and location. Indeed, 18.2% of the children were found to be food-insecure. A significant correlation was evidenced between inadequate consumption of various nutrients below the recommended dietary allowance and food insecurity. Specifically, insufficient protein, vitamin C, fiber, vitamin B12, vitamin B5, vitamin A, vitamin B1, manganese, and copper intake were found to have the highest rates of food insecurity. In addition, children residing in refugee camps experienced significantly higher rates of food insecurity. The findings emphasize the multilayered nature of food insecurity and its impact on children, emphasizing the need for personalized interventions addressing nutrient deficiencies and socioeconomic factors to improve children’s health and well-being.

## 1. Introduction

Food insecurity and poor dietary intake are significant public health concerns affecting millions of children worldwide. The Food and Agriculture Organization (FAO) estimates that over 150 million children suffer from chronic malnutrition, while nearly 2 billion people experience food insecurity around the world [1]. Although food insecurity rates had decreased before the COVID-19 pandemic, they have been continuously on the rise ever since [2,3], highlighting their importance as one of the most significant global public health issues.

Defined as the inability to access sufficient food to meet basic nutritional needs, food insecurity stems from a lack of financial, social, or physical resources [4]. Numerous studies have researched the association of sociodemographic variables and diet on food insecurity [2,5,6,7,8]. Indeed, the literature evidences that the trade-off between less expensive yet less nutritious foods over fruits, vegetables, and other nutrient-dense foods plays a crucial factor among households grappling with food insecurity [9,10,11].

Similarly, extensive research has explored the association between nutrient-specific intake and food insecurity [4,12,13,14,15]. The findings show that a lack of variety in foods consumed, overconsumption of low-nutrient foods, and insufficient food intake all contribute to the prevalence of food insecurity in households [16,17,18]. Furthermore, the studies have found that significant gaps in specific nutrients are present among food-insecure children and adolescents [13].

Both food insecurity and poor nutrient intake are associated with adverse health and behavioral outcomes, including stunted growth, micronutrient deficiencies, obesity, chronic disease prevalence, depression, and low academic performance [12,18,19]. Similarly, research conducted among children and adolescents indicates that food insecurity is associated with lower scores on the Healthy Eating Index (HEI), which indicates inadequate intake of essential nutrients [20,21,22,23].

Food insecurity is, in turn, a key public health concern in Palestine, as 30% of Palestinians in the West Bank experienced food insecurity in 2016, with 9.5% suffering from its most severe form and 7.4% of children facing stunting [24,25]. Expectedly, conflict, agricultural hardships, living areas (such as refugee camps, villages, cities, or proximity to a military checkpoint), and psychological trauma responses (such as overeating unhealthy foods) accentuate a lack of access to food, limit dietary diversity, and overall contribute to food insecurity in the country [25].

Given the complex and multidisciplinary nature of food insecurity, machine learning (ML) algorithms have been used in recent years to predict the association between dietary quality and food insecurity among children and adolescents. The algorithms proposed in the studies have been able to analyse large sets of demographic and dietary data to identify patterns and relationships between variables influencing nutrient intake and food insecurity [14,26,27,28]. The random forest algorithm (RF) has been consistently used to predict food insecurity among children and adolescents. RF uses a decision-tree-based methodology for classifying individuals as either food-secure or food-insecure based on a set of input variables, including household income, household size, and access to food [14,29].

In addition to machine learning algorithms, other predictive models have been developed to identify factors associated with poor dietary quality, such as pre-trained language models for the prediction of nutrition scores [30,31,32,33]. Indeed, machine learning and classification analysis are invaluable for assessing how nutrition is affected by food insecurity, particularly in terms of variations in personalized nutrient intake [24,25,26].

Thus, considering the gaps in research regarding specific nutrient intake and food insecurity in Palestine, this study aims to utilize ML techniques to identify factors associated with food insecurity among Palestinian children and adolescents aged 5 to 18. The study introduces a novel approach by applying ML to explore complex and non-linear relationships in nutritional data. Unlike traditional epidemiological methods, which primarily detect correlations, ML can reveal complicated interactions and patterns that affect nutrient intake and food insecurity. This allows for the creation of predictive models that not only assess risk but also facilitate personalized interventions by identifying individuals at the highest risk. These models enhance the ability to classify varying levels of nutritional health and food security, guiding targeted interventions. Furthermore, machine learning’s capacity to handle high-dimensional data and integrate multiple data types enables a more comprehensive analysis and supports effective, personalized public health strategies. Our innovative approach offers significant advancements over traditional methods by providing actionable insights and tools for addressing nutritional challenges in Palestinian children and adolescents.

## 2. Materials and Methods

### 2.1. Data Source

This study utilized primary data from a food insecurity cross-sectional study that was carried out in the West Bank, Palestine, in 2022. The study assessed the household’s food insecurity, nutritional status, knowledge of nutrition, attitude, and parental practices. The study sample consisted of 1400 households, encompassing a total of 1040 children (aged 5–9 years old) and adolescents (aged 10–18 years old) according to the WHO classifications [34]: 529 from urban areas in the country (cities), 383 from villages, and 128 from refugee camps throughout the West Bank.

This study’s target population consisted of school-aged children and adolescents (≥5 years and <18 years old) in the West Bank. Children and adolescents with disabilities or chronic diseases were excluded from the study. The study variables were collected through a structured, in-person questionnaire. As evidenced in “Supplementary File S1”, the questionnaire encompassed several personal, environmental, and dietary factors that the literature has evidenced as influencing nutrition. Sixteen trained research assistants conducted in-person questionnaires through house visits across West Bank governorates.

This study received ethical approval from the Hebron University ethical committee on 17 October 2022, No. 17/7. Additionally, informed consent was obtained from respondents for all interviews.

### 2.2. Study Variables

The study variables were grouped into four key categories, as described below:

Socioeconomic and Demographic Data: The variables under this section of the questionnaire sought to collect data regarding social and environmental factors that may influence nutrition, including geographical, economic, and household characteristics, as evidenced in Table 1. Parents (usually mothers) were interviewed to provide the household’s socioeconomic and demographic data.

Household Food Insecurity: Food insecurity was assessed using the Radimer/Cornell hunger scale [35,36]. This scale comprises a 10-item questionnaire capturing distinct aspects of food insecurity at the household level, at the individual level for the caregiving adult, and at the individual level for the child.

Nutrition Status (Dietary Intake): A 24 h dietary recall analysis was utilized to assess the nutrient intake of children within the target age range [37]. The method involved parents (mothers) recounting all foods and beverages consumed by their children in the previous 24 h. The tool collected comprehensive data, considering all meals (breakfast, lunch, and dinner) and any snacks consumed, as well as the portion sizes and preparation methods. The 24 h recall method provided a detailed overview of each child’s daily diet.

Anthropometric Measures: Research assistants measured the subject’s height, weight, waist circumference (for children over 12 years old), and upper-mid arm circumference for children below 12 years of age. Height was calculated using a portable SECA body meter with a horizontal headboard attachment. The vertical distance between the standing surface and the top measurement was repeated twice to a precision of 0.1 cm, and a mean value was calculated. The weight was obtained using an SECA digital weighing scale to the nearest 0.1 kg. The respondents were asked to remove their shoes, socks, and heavy clothing.

Waist circumference (WC) was measured as it is an approximate index of intra-abdominal fat. A flexible tape measure was used while participants were asked to stand straight with their arms by their sides. The superior border of the iliac crest and the inferior border of the last rib were marked on both lateral sides of the abdomen. The tape measure was then looped around the waist at the midpoint between the two marks. WC was recorded with a precision of 0.1 cm, and a mean value was calculated.

Similarly, mid-upper arm circumference (MUAC) was calculated for children under 12 years of age. MUAC was recorded to the nearest 0.1 cm using the MUAC measuring tape. Investigators measured MUAC at the midpoint of the arm, where the measuring tape was snugged to the skin. The measurement for each child was taken twice, and the average reading was used as the final measurement. Finally, BMI was calculated based on the International Obesity Task Force (IOTF) definition for children aged 2–18 years [38].

Nutrition Knowledge, Attitudes, and Practices: This section used the Food and Agriculture Organization’s “Knowledge, Attitudes, and Practices” (KAP) questionnaire [1], which sought to collect data related to the individual’s extent of knowledge regarding healthy nutritional practices, their attitudes toward healthy or unhealthy habits, and the extent to which they apply healthy eating practices. A breakdown of variables for each of the aforementioned key sections is illustrated in Table 1.

### 2.3. Nutrient Intake

The 24 h dietary recall data were processed using the EMFID software’s Version 1.0 nutrient analysis tool (EMFID.org, access on 1 September 2021). EMFID is a collaborative food data platform for the Eastern Mediterranean countries, established by Al Quds University in 2021 under the supervision of the World Health Organization (WHO). EMFID’s food composition tables helped to translate the consumed foods and beverages into their corresponding nutritional values. The analysis provided the values of macro- and micronutrient intake, including energy, protein, carbohydrates, fat, B vitamins, vitamin C, and several other minerals, such as magnesium (Mg), potassium (K), phosphorus (P), iron (Fe), and zinc (Zn).

The nutrient intake data were then compared against the Recommended Dietary Allowances (RDAs). RDAs were developed and are updated by the U.S. National Research Council to provide “standards to serve as a goal for good nutrition” [39]. The RDAs determine the specific nutrient requirements for children based on age, gender, and anthropometric measures to assess if the diets meet, exceed, or fall short of the levels recommended.

### 2.4. Machine Learning Methods

In the present study, the Quick, Unbiased, Efficient Statistical Tree (QUEST) algorithm was employed, deriving from the decision tree data mining technique [40], to determine risk factors associated with food insecurity and nutrient intake among Palestinian children and adolescents (5 to 18 years old). The QUEST algorithm is a decision tree algorithm used for classification tasks. It was developed by Loh and Shih [41] to address some of the limitations of earlier algorithms, like CART (classification and regression trees) and C4.5. The main goals of QUEST are to reduce bias in tree splitting, improve computational efficiency, and maintain simplicity in the tree structure [41].

The algorithm produces binary splits at each node, simplifying the tree structure and facilitating its interpretation. QUEST uses statistical tests (like ANOVA for continuous variables and chi-square tests for categorical variables) to select the most optimal split, reducing the bias toward variables encompassing more categories. The algorithm employs a two-step process for splitting: first, it identifies the best variable to split by considering the statistical significance of each variable, and then it determines the best split point for the selected variable.

The splitting process can be described as follows:

For a continuous variable *X*, the algorithm uses the F-statistic from ANOVA to assess the difference in means across groups defined by the target variable *Y*. For a categorical variable, the chi-square statistic is used to assess the association between the variable and the target class.

For the selected variable, the algorithm searches for the split point that maximizes the between-group variance while minimizing the within-group variance (in the case of continuous variables) or maximizes the chi-square statistic (for categorical variables).

Furthermore, the two-stage split decision process of QUEST provides an unbiased approach to determining the best splits in the decision tree. In the first stage, the algorithm conducts either an F-test or a chi-squared test to identify which variable, categorical or numerical, is most significantly associated with the response variable—in this case, food insecurity and dietary quality. This unbiased test stage reduces the risk of bias in split selection.

In the second stage, after a variable type has been selected, the algorithm determines the optimal split for the selected variable type. When a numerical variable, such as ‘energy intake consumed per day’, is selected, a split point is established to amplify the difference between the two groups concerning their food security status and dietary quality. For a categorical variable like education level or refugee status, the algorithm performs an optimal grouping of category levels.

Another unique feature of the QUEST algorithm is its efficiency in dealing with missing values. While other decision tree methodologies might discard such data, QUEST creates surrogate splits. This method helps to maintain the number of data, which is especially crucial in our study considering the potential difficulties in data collection in the context of Palestinian children and adolescents.

Parallel to the QUEST algorithm, the Gini index was utilized to ascertain the importance of each feature in predicting the response variables [42]. The Gini index, a measure of impurity or disorder within a set, aids in determining the optimal splits at each node in the decision-tree-building process. A lower Gini index indicates a “purer” node, with instances primarily belonging to a single class, thereby serving as an effective tool in deciding which features are more critical in predicting the target variables.

## 3. Results

### 3.1. Descriptive Analysis

Table 2 presents the descriptive and univariant analysis carried out to assess the relationship between food security and various sociodemographic and health factors. The results indicate that, among the sample of 1040 children, 18.2% were categorized as food-insecure. Four variables showed a high significance to food insecurity, with family income being the factor that most strongly affects food insecurity, followed by locality, family size, and age.

Family income proved to be a crucial factor in its influence on food insecurity (F = 122.1, *p* = 0.001). The findings show that 60.5% of children in low-income families suffer from food insecurity, compared to 18.7% in middle-income families and 0.7% in high-income families. Similarly, locality is shown to be strongly correlated to food insecurity (F = 68.1, *p* = 0.001). Expectedly, children living in refugee camps exhibit a dramatically high rate of food insecurity (53.1%) compared to children in cities (12.3%) and villages (14.6%).

Family size further shows a significant correlation with food insecurity (F = 9.3, *p* = 0.001) as 25.1% of children in larger families (with over seven members) struggle with food insecurity. In contrast, the prevalence of food insecurity among children in small families (two to four members) is 18.1%, and it is 13.1% for those in families with five to six members. Moreover, a significant correlation was found between food insecurity and age (F = 4.0, *p* = 0.018) as children between the ages of 9 and 12 appeared to be more food-insecure (20.7%), followed by adolescents between 13 and 18 years old (19.9%).

Table 3 presents the detailed associations between food insecurity and nutrient intake. The intake of each nutrient is categorized based on the recommended daily allowance (RDA), with values classified as either above or below the RDA.

Although all nutrients, except for vitamin B1, calcium, and iron, showed statistically significant correlation to food insecurity, this study found that nine nutrients exerted a stronger influence on food insecurity (*p* = 0.001), namely protein, vitamin A, vitamin B12, vitamin B5, vitamin B6, manganese, potassium, copper, and zinc.

Furthermore, an expected pattern emerged whereby higher intake is directly associated with food security, as in the case with energy, protein, carbohydrates, fats, fiber, folate, vitamin A, vitamin B2, vitamin B3, vitamin B5, vitamin B6, vitamin B12, vitamin C, manganese, phosphorous, potassium, copper, and zinc intake, with *p*-values < 0.05. Interestingly, the intake of vitamin B1, calcium, and iron does not significantly differ between the food-secure and food-insecure groups (*p* = 0.171, *p* = 0.261, and *p* = 0.070, respectively), suggesting that these nutrients might not strongly influence food security.

### 3.2. QUEST Model Analysis of Nutritional and Sociodemographic Variables

The decision tree model, evaluated using a 10-fold cross-validation and data split ratios of 70:20:10 for training, testing, and validation, demonstrates robust performance across various metrics. It achieves an AUC of 0.924, indicating strong discriminative ability between classes. The model’s overall accuracy stands at 92.5%, with an F1 score of 0.923, reflecting balanced precision and recall. The precision metric is 0.922, confirming high accuracy when predicting positive cases, while the recall of 0.925 shows effective identification of actual positives. The Matthews correlation coefficient (MCC) of 0.736 further validates the model’s reliability and good predictive quality across different aspects of the data. The model results show that the ML model performs effectively in classifying data with high reliability and accuracy, benefiting from the robustness introduced by cross-validation and careful partitioning of the dataset.

**Figure 1:**
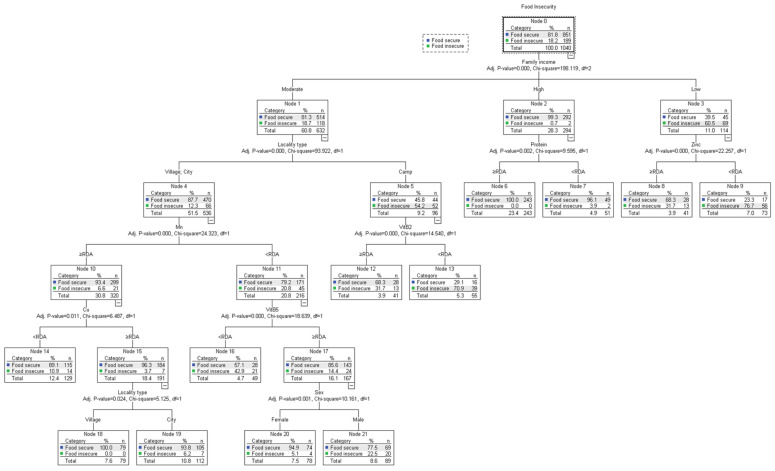
Quick, Unbiased, Efficient Statistical Tree (QUEST) analysis for identifying the pattern of association between food insecurity and study variables.

Figure 1 illustrates the analysis performed by the QUEST algorithm for identifying the pattern of association between sociodemographic and nutrition variables. The analysis evidences that family income is the most significant indicator of food insecurity (χ^2^ = 198.119), with 60.5% of low-income children and adolescents classified as food-insecure. In addition, zinc is most significantly associated with food insecurity (χ^2^ = 22.257) among low-income participants, with 76.7% of those who consume zinc below what the RDA classifies as food-insecure.

Locality type, on the other hand, is most closely associated with food insecurity among middle-income participants (χ^2^ = 93.922), as the majority of food insecurity cases are prevalent among children and adolescents residing in refugee camps (54.2%). In turn, vitamin B2 is shown to play a vital role in contributing to food insecurity for those living in refugee camps (χ^2^ = 14.540), as 70.9% of subjects whose vitamin B2 intake is under the RDA struggle with food insecurity.

In contrast, manganese proved to be associated with food insecurity among those residing in cities and villages (χ^2^ = 24.323), given that 20.8% of those whose Mn intake is below the RDA are classified as food insecure. Consecutively, vitamin B5 is directly associated with food insecurity among the lower Mn intake group (χ^2^ = 18.639), with an FI prevalence of 42% among the vitamin B5 intake group below the RDA. Affecting food insecurity among the vitamin B5 intake group < RDA, gender (χ^2^ = 10.161) plays a significant role as FI prevalence is evidently higher among males (22.5%) than females (5.1%).

The factors associated with food insecurity among those residing in cities and villages with a Mn intake at or above the RDA encompassed copper (χ^2^ = 6.487) and locality type (χ^2^ = 5.125) (among the Cu ≥ RDA group). Indeed, lower copper intake signified higher FI prevalence (10.9%), while those living in cities among the higher copper intake group were slightly more likely to be food-insecure (6.2%) than those residing in villages.

Finally, one factor showed a significant association with FI among participants from high-income households, namely protein (χ^2^ = 9.595), as lower consumption of protein shows a slightly higher prevalence of food insecurity (3.9%) than those whose protein intake is at or above RDA (0%).

### 3.3. QUEST Model Analysis of Nutritional Variables

**Figure 2:**
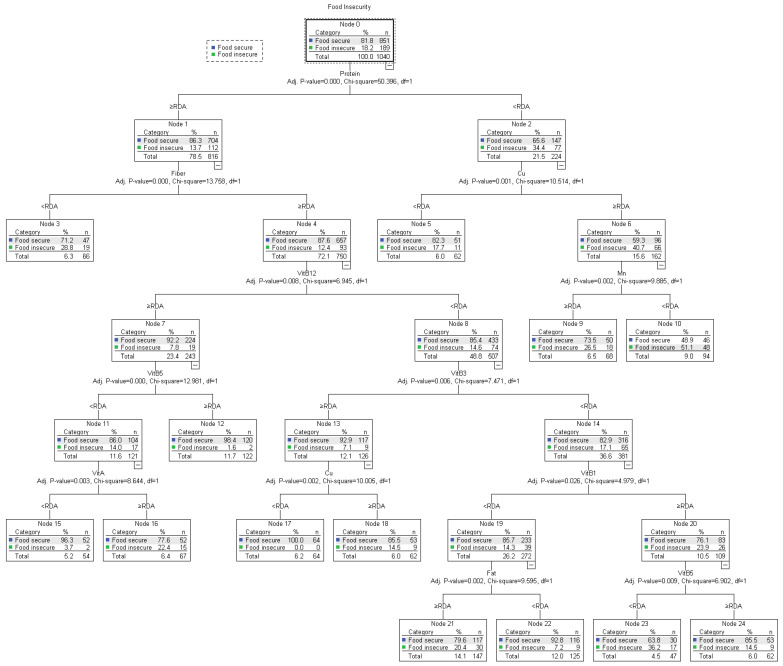
Quick, Unbiased, Efficient Statistical Tree (QUEST) analysis for identifying the pattern of association between food security and nutrient intakes.

The results in Figure 2 illustrate the QUEST analysis, which identified the association between food insecurity and children’s nutrient intake. When considering only vitamins and minerals in the QUEST algorithm, the analysis evidences that protein is most significantly associated with food insecurity (χ^2^ = 50.396). Expectedly, lower protein intake is associated with higher FI prevalence (34.4%). Among the low protein intake group, copper (χ^2^ = 10.514), followed by manganese, showed a close association with food insecurity. Interestingly, higher copper intake is associated with higher FI prevalence (40.7%), which is in turn associated with manganese intake (χ^2^ = 9.885), as higher food insecurity is prevalent among the lower Mn intake group.

Among the high protein intake group, fiber proves a key association (χ^2^ = 13.758), as lower fiber intake is associated with higher food insecurity rates (28.8%). In turn, vitamin B12’s direct association with the higher fiber intake group (χ^2^ = 6.945) is evidenced, as lower vitamin B12 intake is correlated with higher food insecurity (14.6%). Indeed, lower Vit B12 shows an association with vitamin B3 (χ^2^ = 7.471), as 17.1% of participants with lower Vit B3 intake were classified as food-insecure. In turn, vitamin B1 (χ^2^ = 4.979), vitamin B5 (χ^2^ = 6.902), and fat (χ^2^ = 9.595) showed direct associations to food insecurity among the Vit B3 intake group < RDA. Within this category, higher Vit B1 intake shows higher FI prevalence (23.9%), which is directly associated with Vit B5, as lower Vit B5 intake shows higher FI rates (36.2%). Finally, fat shows a direct association with lower vitamin B1 intake, as higher fat intake presents a higher prevalence of food insecurity (20.4%).

Lastly, among the higher vitamin B12 intake group, vitamin B5 and vitamin A showed significant associations (χ^2^ = 12.961 and χ^2^ = 8.644, respectively). While lower Vit B5 intake presented higher FI rates (14%), higher Vit A intake is associated with higher FI prevalence among the Vit B5 ≥ RDA category (22.4%).

### 3.4. Gini Coefficient Importance Analysis

**Figure 3:**
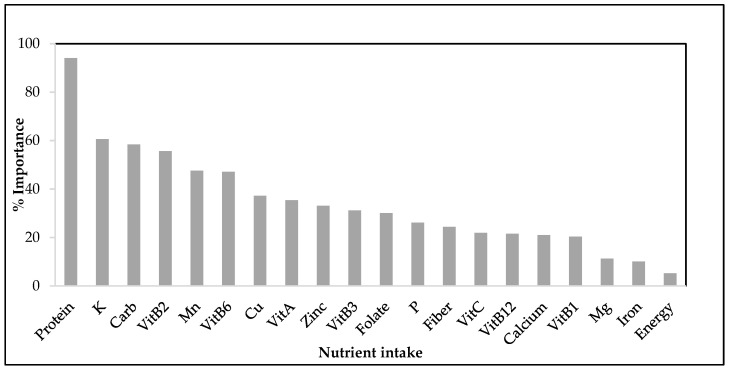
Gini importance analysis of nutrient intake against food security among children and adolescents aged 5–18 years.

Furthermore, an analysis using Gini importance was performed to identify the factors most significantly associated with food insecurity among children and adolescents. The variables yielding the highest scores in the analysis are thus considered the most significant indicators, as illustrated in Figure 3.

The results depicted in Figure 3 present the normalized importance scores obtained from the random forest model, which observed nutrient intake as a factor. The results evidence that nutrients such as protein, potassium, carbohydrates, vitamin B12, manganese, vitamin B6, copper, vitamin A, zinc, and folate, reporting importance levels > 30%, are most strongly associated with food security.

## 4. Discussion

The study results underline the important relationship between dietary nutrient intake and sociodemographic factors, such as age, gender, income, and location, concerning food security, in children and adolescents aged 5 to 18 years. The findings offer a comprehensive view of the multifaceted nature of food insecurity given its correlation to sociodemographic and health factors among children and adolescents.

Several analyses of the data were conducted, including univariate, QUEST, and GINI importance analyses. The univariate and QUEST analyses were duplicated by first encompassing sociodemographic variables and then by limiting the variables to vitamins and minerals to assess the specific correlation among nutrients. Indeed, both the univariate and QUEST analyses found that family income, locality (city, village, or refugee camp), and family size were the most statistically significant factors influencing food insecurity among Palestinian children and adolescents. Unexpectedly, the study did not find an association between food insecurity, gender, and BMI.

The results are consistent with previous studies, such as [9,43,44], whereby family income and family size emerged as significant determinants of food insecurity. Similarly, the findings align with [27,45,46], evidencing the association between age and food insecurity as increased nutritional needs among older children might increase FI prevalence. Furthermore, the high prevalence of food insecurity in refugee camps is consistent with global reports, such as those by the FAO, IFAD, UNICEF, WFP, and WHO, that mention the elevated risk of food insecurity among displaced populations living in refugee camps [1]. The above results emphasize the urgent need for targeted food security interventions among lower-income households with more members, within older age groups, and in refugee camps.

Interestingly, the study found no significant correlation between sex or BMI and food insecurity, aligning with previous studies that found no meaningful difference in food insecurity based on gender [9,22,47]. This suggests that food insecurity is not gender-discriminatory at the child level, highlighting the need for interventions that target all children, irrespective of their gender. Nonetheless, the lack of association between BMI and food security contrasts with several studies indicating that children with low BMI often face higher rates of food insecurity [9,38,48]. The contrasting findings indicate the need for further research in different contexts to more closely examine this relationship.

Similarly, the nutrient analysis in the univariate, QUEST, and GINI analyses found that protein intake is more significantly associated with food insecurity than other nutrients, which is consistent with the results of previous studies [49], which revealed protein intake as a significant determinant of food security. Other significant associations were found, including vitamin A, vitamin B12, vitamin B5, manganese, potassium, copper, and zinc, aligning with previous research [22] that suggests that children facing food insecurity tend to consume diets lower in caloric content and essential macronutrients.

Interestingly, no significant difference was found in vitamin B1 and magnesium intake between the food-secure and food-insecure groups. This contrasts with previous research, where food-insecure individuals had lower nutrient intakes across the board. The lack of a significant difference for these specific nutrients in our study may be due to a variety of factors, such as a limited sample size, geographic location, or access to specific nutrient-rich foods [16,50]. Further investigation is required to better understand this observation.

Notably, a significant relationship between Mn intake and food security was evidenced across the board. Although Mn is a trace mineral and is required in smaller amounts than other nutrients, this finding suggests that it plays a vital role in food security, likely due to its involvement in several physiological functions [51]. Furthermore, the lack of food insecurity prevalence among those meeting the RDA for copper (Cu) is a unique observation that merits further investigation. Although Cu is a trace mineral, its role in various physiological functions might be crucial for maintaining food security [51].

Furthermore, the QUEST analysis evidenced patterns among intake levels of certain nutrients leading to higher or lower instances of food insecurity. Notably, such patterns will support the development of more personalized nutritional interventions among at-risk populations. This analysis further emphasizes the role of a balanced, nutrient-rich diet in maintaining food security among children. While meeting RDAs for specific nutrients seems to be associated with higher food security, some level of food insecurity persists even when RDAs are met. This points to the fact that food security is influenced by an array of factors beyond nutrient intake alone. As per [52], factors such as socio-economic status, food access, and overall diet quality also play a significant role in food security.

## 5. Strengths and Limitations

This study possesses several strengths that enhance its significance and validity. Firstly, it conducts a comprehensive analysis that explores the relationship between dietary nutrient intake and sociodemographic factors, such as age, gender, income, and location, concerning food security among children aged 5 to 18 years. By considering multiple factors, the study provides a comprehensive understanding of the intricate dynamics influencing food security in this population. The study also demonstrates methodological innovation by incorporating several methods for statistical analysis, including advanced machine learning techniques such as the QUEST analysis and Gini importance analysis. These techniques offer a fresh framework for comprehending the intricate interplay between nutrient intake, food security, and sociodemographic factors. By utilizing these sophisticated analytical approaches, the study deepens the analysis and provides valuable insights into the complex relationships among these factors.

However, it is important to consider the limitations of this study. Despite the algorithm’s effectiveness, it is worth noting the limitations of the QUEST algorithm, as it only supports binary splits and has a relatively more complex structure than other decision trees such as CART or ID3. This complexity may be a disadvantage in scenarios where ease of interpretability is crucial. Furthermore, while its method for handling missing values is a benefit, it could lead to a more intricate tree structure if the data have a substantial number of missing values. Moreover, the cross-sectional design employed restricts the ability to establish causal relationships between nutrient intake, sociodemographic factors, and food security. Longitudinal studies would be needed to provide more robust evidence regarding the temporal dynamics of these associations. Additionally, relying on self-reported data for nutrient intake and food security status introduces the potential for recall bias and social desirability bias, which could impact the accuracy of the reported information.

Furthermore, the generalizability of this study’s findings may be constrained to the specific context of Palestinian children. Caution should be considered when extrapolating the results to other populations or regions with different sociocultural and economic backgrounds. Lastly, the reliability and validity of this study’s findings are contingent upon the quality and availability of the collected data, including any potential limitations or missing data in the data collection methods.

## 6. Conclusions

This study investigated the complexity of factors that contribute to food security, including the interactions between multiple nutrients, socio-demographic variables, and other health determinants. While this research offers valuable insights on the impact of food insecurity on children and adolescents’ nutrient intake, the model identified a significant association with protein, vitamin A, vitamin B12, vitamin B5, manganese, potassium, copper, and zinc. Aligning with previous research, it also emphasizes the importance of continued research efforts. Thus, this study indicated the importance of comprehensive nutritional support in tackling food insecurity. Policymakers and practitioners should consider interventions that not only provide sufficient food but also ensure a well-balanced diet that meets the RDAs for key nutrients while taking into consideration the patterns of association found in this study.

## Figures and Tables

**Table 1:** Study variables.

Section	Items
Socioeconomic and Demographic Data	Gender, age, governorate, living area (south, middle, and north), locality (refugee camp, village, or city), education level, family size, father’s employment, mother’s employment, family income.
Household Food Insecurity	Food quantity, food quality, food acceptability, and the certainty of obtaining food.
Nutrition Status (Dietary Intake)	24 h recall: grams intake, calories, protein, carbs, fiber, cholesterol, vitamin B1, vitamin B2, vitamin B3, vitamin B5, vitamin B6, choline, folate, vitamin B12, vitamin C, vitamin E, vitamin K1, calcium, chloride, magnesium, phosphorous, potassium, sodium, copper, fluoride, iron, manganese, zinc.
Nutrition Status (Anthropometric Measures)	Height, weight, mid-upper arm circumference, waist circumference, body mass index (BMI).
Nutrition-related Practices	Difficulty eating breakfast; difficulty eating three meals.

**Table 2:** Food insecurity and its association with sociodemographic and health factors among Palestinian children and adolescents in the West Bank.

Variable	Overall Food Security		
	Food Security *n* (%)	Food Insecurity *n* (%)	F (*p*-Value)
**Total**	**851 (81.8)**	**189 (18.2)**	
**Gender**			
Male	399 (78.5)	109 (21.5)	7.2
Female	452 (85)	80 (15)	
**Age (Year)**			
5–8	263 (87.1)	39 (12.9)	4.0 *
9–12	287 (79.3)	75 (20.7)	
13–18	301 (80.1)	75 (19.9)	
**Living Area**			
South	339 (81.7)	76 (18.3)	0.290
Middle	279 (80.9)	66 (19.1)	
North	233 (83.2)	47 (16.8)	
**Locality**			
City	464 (87.7)	65 (12.3)	68.1 **
Village	327 (85.4)	56 (14.6)	
Camp	60 (46.9)	68 (53.1)	
**Family Income**			
Low	45 (39.5)	69 (60.5)	122.1 **
Moderate	514 (81.3)	118 (18.7)	
High	292 (99.3)	2 (0.7)	
**Family Size**			
2–4	213 (81.9)	47 (18.1)	9.3 **
5–6	390 (86.9)	59 (13.1)	
7+	248 (74.9)	83 (25.1)	
**BMI**			
Underweight	374 (81.3)	86 (18.7)	0.303
Normal	317 (81.3)	73 (18.7)	
Overweight	115 (83.9)	22 (16.1)	
Obese	45 (84.9)	8 (15.1)	

Descriptive and univariant analysis: * *p* < 0.05; ** *p* < 0.001.

**Table 3:** Association between food insecurity and nutrient intake among Palestinian children and adolescents in the West Bank.

Nutrient (Unit)	Nutrient Intake per RDA		Food Security Level		
	≥RDA *n* (%)	<RDA *n* (%)	Food-Secure Mean ± SD	Food-Insecure Mean ± SD	F (*p*-Value)
Energy (kcal)	768 (73.8)	272 (26.2)	1584.4 ± 616.4	1465.8 ± 813	4.8 *
Protein (g)	816 (78.5)	224 (21.5)	58.7 ± 27.3	52.7 ± 34	47.2 **
Carb (g)	842 (81)	198 (19)	216.1 ± 91.9	203.8 ± 115.7	8.3 *
Fat (g)	517 (49.7)	523 (50.3)	55.7 ± 27.3	51.2 ± 30.7	7.6 *
Fiber (g)	74 (7.1)	966 (92.9)	13.5 ± 7.8	13.8 ± 9.9	9 *
Folate (mg)	760 (73.1)	280 (26.9)	177.5 ± 115.4	151.4 ± 121	9.4 *
Vit A (UI)	745 (71.6)	295 (28.4)	134.2 ± 136	126 ± 125.2	12.3 **
VitB1 (mg)	312 (30)	728 (70)	1.4 ± 2.1	1.6 ± 2.4	1.8
VitB2 (mg)	529 (50.9)	511 (49.1)	2.6 ± 3.7	2.2 ± 4.8	19.3 **
VitB3 (mg)	276 (26.5)	764 (73.5)	9.7 ± 7.7	8.1 ± 7.3	9.8 *
VitB5 (mg)	397 (38.2)	643 (61.8)	3.4 ± 2.1	3.3 ± 2.4	19.2 **
VitB6 (mg)	743 (71.4)	297 (28.6)	2.4 ± 3.8	2.4 ± 4	12.4 **
VitB12 (mcg)	288 (27.7)	752 (72.3)	2.3 ± 4.9	2.4 ± 5.5	5.7 *
Vit C (mg)	225 (21.6)	815 (78.4)	60.7 ± 62.4	43.7 ± 52.1	6.4 *
Ca ^1^ (mg)	31 (3)	1009 (97)	473 ± 289.5	401.4 ± 381	2.9
Mg ^2^ (mg)	201 (19.3)	839 (80.7)	155.6 ± 81.5	163.5 ± 122.9	1.2 *
Mn ^3^ (mg)	636 (61.2)	404 (38.8)	2.1 ± 3.5	2.3 ± 3.7	16.7 **
P ^4^ (mg)	235 (22.6)	805 (77.4)	691.7 ± 333.8	670.6 ± 485.5	9.2 *
K ^5^ (mg)	6 (0.6)	1034 (99.4)	1361.1 ± 673.2	1272 ± 908.2	27.8 **
Cu ^6^ (mg)	445 (42.8)	595 (57.2)	2.1 ± 2.8	2.1 ± 3.3	12.8 **
Fe ^7^ (mg)	383 (36.8)	657 (63.2)	9.6 ± 6	8.8 ± 5.7	3.3
Zn ^8^ (mcg)	354 (34)	686 (66)	7.2 ± 5.5	6.3 ± 5.4	12.0 **

* *p* < 0.05; ** *p* < 0.001; ^1^ Ca = calcium, ^2^ Mg = magnesium, ^3^ Mn = manganese, ^4^ P = phosphorous, ^5^ K = potassium, ^6^ Cu = copper, ^7^ Fe = iron, ^8^ Zn = zinc.

## Data Availability

The data that support the findings of this study are available on request from the corresponding author. The data are not publicly available due to their ongoing use by other researchers who are preparing additional publications.

## Supplementary Materials

The following supporting information can be downloaded at: https://www.mdpi.com/article/10.3390/children11060625/s1. File S1: Association of Household Food Insecurity with Nutritional Status and Nutrition-Related Knowledge, Attitudes, and Practices among Various Age Groups in the West Bank, Palestine.
