# Supplementary material for: Investigating the Association between Nutrient Intake and Food Insecurity among Children and Adolescents in Palestine Using Machine Learning Techniques
Source: Children (Basel). 2024 May 23;11(6):625. doi: 10.3390/children11060625 (PMC11202236; doi:10.3390/children11060625)
Supplement: Supplementary file 1 [file children-11-00625-s001.zip › children-2922563-supplementary.pdf]

<u>Questionnaire</u>

Association of Household Food Insecurity with Nutritional Status and Nutrition-Related Knowledge, Attitudes, and Practices among Various Age Groups in the West Bank, Palestine

Dear participant

You are invited to join the above-mentioned research study. You have been selected as a participant because you meet the participation criteria. This study aims to: (1) identify the level of household food insecurity in West Bank; (2) assess nutrition-related knowledge, attitudes and practices (KAP) in various age groups; (3) investigate the association of households food insecurity with dietary intakes, anthropometric measures and nutrition-related KAP of various age groups; and (4) examine the association of nutrition-related KAP with dietary intakes and anthropometric measures of various age groups in West Bank governorates.

The interview will take about 30 minutes, all the information we obtain will be kept strictly confidential and your answers and your name will not be revealed, and no information that would enable the identification of any participant will be published. Also, you don't have to answer any question you don't want to, and you can stop the interview at any time.

I would like you to answer the questions honestly, feel free to answer questions at your own pace.

Do you agree to voluntarily participate in this interview? □ Yes □ No

If the answer is yes, continue to the next question. If the answer is no, stop the interview.

Do you have any question before we start?

Can I start now?

We thank you for your kind cooperation and appreciate your positive decision to participate in the research

Socio-Economic and Demographic Characteristics of the Households		
**1.**	**Name**	………………………
**2.**	**Gender**	□ Male □ Female
**3.**	**Age (year)**	……………………….
**4.**	**Governorate**	□ North west bank □ south west bank □ Middle Area
**5.**	**The nature of the living area**	□ City □ Village □ Camp □ Other..........................
**6.**	**Educational level**	□ Illiterate □ Primary □ Prep □ Secondary □ University
**7.**	**Number of family members**	………………………
**8.**	**Family income (NIS)**	□ > 1000 □ 1000-2000 □ 3000 - 2001 □ < 3000
**9.**	**Does the family receive food aid?**	□ Yes □ No
**10.**	**Home ownership**	□ Owned □ Rented
**11.**	**Work status**	□ Has work □ Do not has work
**12.**	**Marital Status**	□ Single □ Married □ Divorced □ widowed

Radimer / Cornell Hunger and Food Insecurity Items

No.	Question	Not True	Sometimes True	Always True
	**A. Household level**			
**1.**	I worry that if the food or raw materials for cooking will run out before I could have more money to buy food.			
**2.**	Food or raw materials for cooking that I bought for my family at home is always run out fast and I do not have the money to buy food again.			
**3.**	I do not have enough food or raw materials to cook or prepare a family meal (for the tomb of the morning, noon or night) and I did not have enough money to buy food.			
**4.**	We eat the same thing for several days in a row because we only have a few different kinds of food on hand and do not have money to buy more.			
	**B. Individual level (Adult)**			
**5.**	I am often hungry but I do not eat because I do not have enough money to buy food.			
**6.**	I only eat a little of what I should eat because I do not have enough money to buy food.			
**7.**	I was not able to eat properly or eat to satiety because I do not have enough money to buy food.			
	**C. Individual level (Child)**			
**8.**	I am not able to provide a balanced meal to my children because I do not have enough money to provide food.			
**9.**	My children do not eat enough or always lack of food because I am not able to buy enough food.			
**10.**	I know sometimes my children are hungry, but I cannot do anything because I am not able to buy food in excess of what I always buy			

For Under Five Children		
**Background**		
**1.**	**Name**	……………………………………….
**2.**	**Gender**	□ Male □ Female
**3.**	**Date of birth**	……………………………………….
**4.**	**Age (Year)**	……………………………………….
**5.**	**Gestational age (Weeks)**	……………………………………….
**6.**	**Governorate**	□ North west bank □ south west bank □ Middle Area
**7.**	**The nature of the living area**	□ City □ Village □ Camp □ Other..........................
**Medical history**		
**8.**	**Does the child has any disease?**	□ Yes □ No
If Yes, Determine		
**9.**	**During pregnancy, did mother has gestational diabetes?**	□ Yes □ No
**10.**	**Does the child take any medication?**	□ Yes □ No
If Yes, Determine		

Anthropometric measures				
**No.**	**Measure:**	**First measure**	**Second measure**	**Mean**
**1.**	Weight (kg)			
**2.**	Height/length (cm)			
**3.**	Mid Upper Arm Circumference (cm)			
**4.**	Birth weight (kg)			

**KAP about feeding young children (6–23 months)**			
**Knowledge**			
**Continued breastfeeding**			
**1. Probe if necessary: Until what age is it recommended that a mother continues breastfeeding?**			
□ Six months or less	□ 11 months	□ 12-23 months	□ 24 months and more
□ Other	□ Don't know		
**Age of start of complementary foods**			
**2. At what age should babies start eating foods in addition to breast milk?**			
□ At six months	□ Other	□ Don't know	
**Reason for giving complementary foods at six months**			
**3. Why is it important to give foods in addition to breast milk to babies from the age of six months?**			
□ Breast milk alone is not sufficient (enough)/cannot supply all the nutrients needed for growth/from six months, baby needs more food in addition to breast milk	□ Other	□ Don't know	
**Consistency of meals**			
**4. Please look at these two pictures of porridges. Which one do you think should be given to a young child?** (Show the images/pictures of thick and watery/thin porridges and tick one of the options here below depending on the respondent answer).			
□ Shows the thick porridge	□ Shows the watery	□ Does not know	
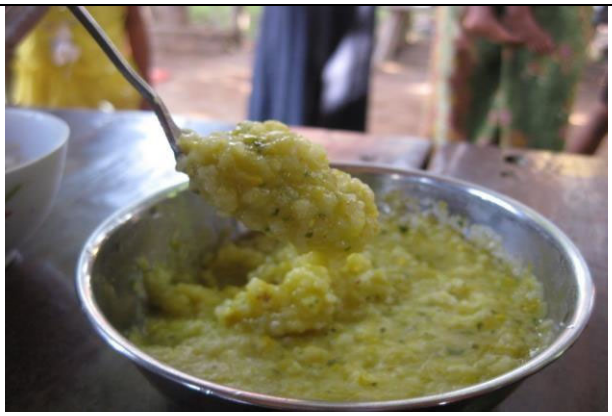	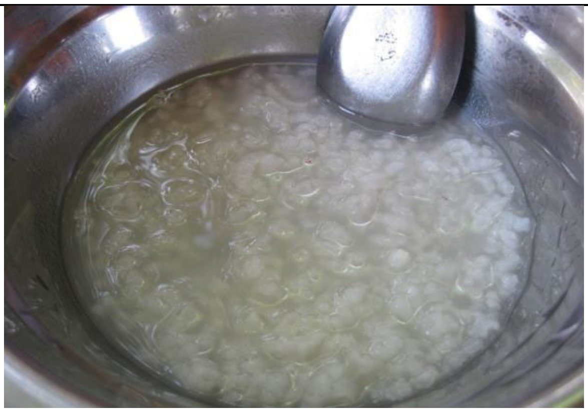		

**Reason for consistency of meals**
**5. Why did you pick that picture?**
□ Because the first porridge is thicker than the other<br>□ Because the thick porridge is more nutritious/because it is prepared with different types of foods or ingredients (food diversity) □ Other □ Don't know
**Dietary diversity and ways of enriching porridge**
To feed their children, many mothers give them rice porridge or borbor. Please tell me some ways to make rice porridge more nutritious or better for your baby's health. Probe if necessary:<br>**6. Which foods or types of food can be added to rice porridge make it more nutritious? By adding:**
□ Animal-source foods (meat, poultry, fish, liver/organ meat, eggs, etc.)<br>□ Pulses and nuts: flours of groundnut and other legumes (peas, beans, lentils, etc.), sunflower seed, peanuts, soybeans<br>□ Vitamin-A-rich fruits and vegetables (carrot, orange-fleshed sweet potato, yellow pumpkin, mango, papaya, etc.)<br>□ Green leafy vegetables (e.g. spinach) □ Energy-rich foods (e.g. oil, butter/ghee)<br>□ Other □ Don't know
**Responsive feeding**
**7. Do you know any ways to encourage young children to eat?**
□ Giving them attention during meals, talk to them, make meal times happy times □ Clap hands<br>□ Make funny faces/play/laugh □ Demonstrate opening your own mouth very wide/modeling how to eat<br>□ Say encouraging words □ Draw the child's attention □ Other □ Don't know
**Attitudes towards an ideal or desired nutrition-related practice**
**Self-confidence**
**8. How confident do you feel in preparing food for your child?**
□ Not confident □ Ok/so-so □ Confident
**Giving a diversity of food (foods from many food groups (**
**Perceived benefits**

**9. How good do you think it is to give different types of food to your child each day?**
□ Not good □ You're not sure □ Good
**Perceived barriers**
**10. How difficult is it for you to give different types of food to your child each day?**
□ Not difficult □ So-so □ Difficult
**Feeding frequently**
**Perceived benefits**
**11. How good do you think it is to feed your child several times each day?**
□ Not good □ You're not sure □ Good
**Perceived barriers**
**12. How difficult is it for you to feed your child several times each day?**
□ Not difficult □ So-so □ Difficult
**Continuing breastfeeding beyond six months**
**Perceived benefits**
**13. How good do you think it is to continue breastfeeding beyond six months?**
□ Not good □ You're not sure □ Good
**Perceived barriers**
**14. How difficult is it for you to continue breastfeeding beyond six months?**
□ Not difficult □ So-so □ Difficult
**Practices**
**Continued breastfeeding**
**15. Was (name of the baby) breastfed or did he or she consume breast milk yesterday during the day or at night?**
□ Yes □ No □ Don't know/no answer

**Dietary diversity**
Now I would like to ask you about (other) liquids or foods that (name of the baby) ate yesterday during the day or at night. I am interested in whether your child had the item even if it was combined with other foods. For example, if (name of the baby) ate a millet porridge made with a mixed vegetable sauce, you should reply yes to any food I ask about that was an ingredient in the porridge or sauce. Please do not include any food used in a small amount for seasoning or condiments (like chillies, spices, herbs or fish powder); I will ask you about those foods separately.<br>**Yesterday during the day or at night, did (name of the baby) eat:**<br>Read the food lists. Underline the corresponding foods consumed and tick the column Yes or No depending on whether any food item of the list was consumed. **Record the number of times when relevant (Group 3).**

Group	Food lists	No	Yes
**Group 1**:<br>Grains, roots and tubers	Porridge, bread, rice, noodles or other foods made from grains		
	White potatoes, white yams, manioc, cassava or any other foods made from roots		
**Group 2**: Legumes and nuts	Any foods made from beans, peas, lentils, nuts or seeds		
**Group 3**:<br>Dairy products	Infant formula<br>How many times? ………………….		
	Milk, such as tinned, powdered or fresh animal milk<br>How many times? ………………….		
	Yogurt<br>How many times? ………………….		
	Cheese or other dairy products		
**Group 4**:<br>Flesh foods	Liver, kidney, heart or other organ meats		
	Any meat, such as beef, pork, lamb, goat, chicken or duck		
	Fresh or dried fish, shellfish or seafood		

	Grubs, snails or insects		
**Group 5**: Eggs	Eggs		
**Group 6**:<br>Vitamin A fruits and vegetables	Pumpkin, carrots, squash or sweet potatoes that are yellow or orange inside		
	Any dark green vegetables		
	Ripe mangoes (fresh or dried [not green]), ripe papayas (fresh or dried), musk melon **[insert other local vitamin-A-rich fruits]**		
	Foods made with red palm oil, red palm nut or red palm nut pulp sauce		
**Group 7**:<br>Other fruits and vegetables	Any other fruits or vegetables		
**Others**<br>(not counted in the dietary diversity score)	Any oil, fats, or butter or foods made with any of these		
	Any sugary foods, such as chocolates, sweets, candies, pastries, cakes or biscuits		
	Condiments for flavour, such as chillies, spices, herbs or fish powder		

**Minimum meal frequency**	
**How many times did (name of the baby) eat foods, that is meals and snacks other than liquids yesterday during the day or at night?**	□ Number of times ……………<br>□ Don't know/no answer

First day: 24-hour dietary recall

Determine what was yesterday:

□ Saturday	□ Sunday	□ Monday	□ Tuesday	□ Wednesday	□ Thursday	□ Friday

Would you describe the food that your child ate yesterday as atypical of your habitual food intake? □ Yes □ No

No.	Food Name	Time	Occasion (Breakfast – Launch – Dinner –Other)	Source of food (Homemade – Restaurant – Supermarket – Other)	Amount (According To Kitchen Staff)

Second day: 24-hour dietary recall

Determine what was yesterday:

□ Saturday	□ Sunday	□ Monday	□ Tuesday	□ Wednesday	□ Thursday	□ Friday

Would you describe the food that your child ate yesterday as atypical of your habitual food intake? □ Yes □ No

No.	Food Name	Time	Occasion (Breakfast – Launch – Dinner –Other)	Source of food (Homemade – Restaurant – Supermarket – Other)	Amount (According To Kitchen Staff)

Socio-demographic questionnaire for school-aged children (5-18 years old)		
**1.**	**Child name**	…………………………………….
**2.**	**Sex**	□ Male □ Female
**3.**	**Age (years)**	…………………………………….
**4.**	**Governorate**	□ North west bank □ south west bank □ Middle Area
**5.**	**Where do you live?**	□ City □ Village □ Refugees camp □ Other
**6.**	**Educational level**	□ Primary school □ Preparatory school □ Secondary school

Anthropometric measures		
**1.**	Height (cm)	…………………………………….
**2.**	Weight (kg)	…………………………………….
**3.**	Mid Upper Arm Circumference (cm)	…………………………………….

KAP about: Diet of school-aged children (5-18 years old)
**Knowledge**
**Consequences of short-term hunger at school**
**1. What problems can children have if they do not eat before going to school?**
□ Children have short attention/have low concentration/cannot study well/do not do as well at school as they should<br>□ Other □ Don’t know
**Discouraging sweets and candies**
**2. Why is it so bad to eat too many sweets and candies?**
□ Because they can cause tooth decay □ Because they are not nutritious □ Because they interfere with appetite<br>□ Other □ Don’t know
**Attitudes towards an ideal or desired nutrition-related practice**
**Attitudes**

**Having breakfast before going to school**		
**Perceived benefits**		
**3. How good do you think it is to have breakfast before going to school?**		
□ Not good	□ You're not sure	□ Good
**Perceived barriers**		
**4. How difficult is it for you to have breakfast before going to school?**		
□ Not difficult	□ So-so	□ Difficult
**Having three meals a day and snacks**		
**Perceived benefits**		
**5. How good do you think it is to have three meals a day and snacks ?**		
□ Not good	□ You're not sure	□ Good
**Perceived barriers**		
**6. How difficult is it for you to have three meals a day and snacks?**		
□ Not difficult	□ So-so	□ Difficult
**Having different types of foods at meal times**		
**Perceived benefits**		
**7. How good do you think it is to have different types of foods at meals ?**		
□ Not good	□ You're not sure	□ Good
**Perceived barriers**		
**8. How difficult is it for you to have different types of foods at meals?**		
□ Not difficult	□ So-so	□ Difficult

**Practices**
**Having breakfast: time and place**
**9. Did you have breakfast before going to school?**

□ Yes	□ No	□ Don’t know/no answer
**10. If Yes: At what time?**		
□ Between 6 a.m. and 9 a.m	□ Between 9 a.m. and noon	□ Between noon and 3 p.m
**11. Where?**		
□ Home	□ School	□ Elsewhere
**Having lunch: time and place**		
**12. If the interview is being conducted before lunchtime, ask: Did you have lunch yesterday? If the interview is being conducted after lunchtime, ask: Did you have lunch today?**		
□ Yes	□ No	□ Don’t know/no answer
**13. If Yes: At what time?**		
□ Between 9 a.m. and noon	□ Between noon and 3 p.m	□ Between 3 p.m. and 6 p.m
**14. Who prepares your lunch?**		
□ Parents at home<br>□ Lunch is bought with pocket money	□ School cafeteria<br>□ Other	
**Dinner/supper: time and place**		
**15. Did you have dinner yesterday?**		
□ Yes	□ No	□ Don’t know/no answer
**16. If Yes: At what time?**		
□ Between 3 p.m. and 6 p.m	□ Between 6 p.m. and 9 p.m	□ Between 9 p.m. and midnight
**17. Where?**		
□ Home	□ School	□ Elsewhere
**Snacks**		
**18. Yesterday during the day and night, did you eat anything between the meals?**		
□ Yes	□ No	□ Don’t know/no answer
**Bought food**		

**19. Yesterday during the day and night, did you buy foods with your own money?**		
□ Yes	□ No	□ Don’t know/no answer
**20. Where did you buy those foods?**		
□ At school/cafeteria	□ On the street (from street vendors)	□ Other

First day: 24-hour dietary recall

Determine what was yesterday:

□ Saturday	□ Sunday	□ Monday	□ Tuesday	□ Wednesday	□ Thursday	□ Friday

Would you describe the food that your child ate yesterday as atypical of your habitual food intake? □ Yes □ No

No.	Food Name	Time	Occasion (Breakfast – Launch – Dinner –Other)	Source of food (Homemade – Restaurant – Supermarket – Other)	Amount (According To Kitchen Staff)

Second day: 24-hour dietary recall

Determine what was yesterday:

□ Saturday	□ Sunday	□ Monday	□ Tuesday	□ Wednesday	□ Thursday	□ Friday

Would you describe the food that your child ate yesterday as atypical of your habitual food intake? □ Yes □ No

No.	Food Name	Time	Occasion (Breakfast – Launch – Dinner –Other)	Source of food (Homemade – Restaurant – Supermarket – Other)	Amount (According To Kitchen Staff)

Socio-demographic questionnaire for adults (> 18 years)		
**1.**	**Name**	…………………………………………….
**2.**	**Sex**	□ Male □ Female
**3.**	**Age (year)**	…………………………………………….
**4.**	**Governorate**	□ North West Bank □ Middle Area □ South West Bank
**5.**	**The nature of the living area**	□ City □ Village □ Camp □ Other
**6.**	**Educational level**	□ Illiterate □ Primary □ Prep □ Secondary □ University
**Anthropometric measurements**		
**1.**	**Height (meter)**	…………………………………………….
**2.**	**Weight (kg)**	…………………………………………….
**3.**	**Waist circumference (cm)**	…………………………………………….

KAP: Undernutrition

Knowledge

1. What are the signs of undernutrition?

□ Lack of energy / weakness: cannot work, study or play as normal (disability).

□ Weakness of the immune system (becomes ill easily or becomes seriously ill).

□ Loss of weight / thinness.

□ Children do not grow as they should (growth faltering).

□ Other: ………………………………………….. □ Don't know

2. What are the reasons why people are undernourished?

□ Not getting enough food □ Food is watery, does not contain enough nutrients

□ Disease / ill and not eating food □ Other □ Don't know

3. What are the reasons why people do not get enough food?

□ Not having enough money to buy food □ Food is not available □ Other □ Don't know

**4. Who can help the mother to find out if the baby is growing well? Where can she go?**<br>□ Go to the health center / ask a doctor or nurse (health professional) (seeking health-care services for growth monitoring). □ Other □ Don't know
**5. If the baby is not gaining weight, what does that mean? What could be the causes?**<br>□ The baby is not eating well/the baby does not want to eat □ The baby may be sick often<br>□ Other □ Don't know
**6. What should we do to prevent undernutrition among infants (0–6 months)?**<br>□ Breastfeed exclusively / give only breast milk □ Other □ Don't know<br>□ Go to the health center / hospital and check that the child is growing (growth monitoring services).
**7. What should we do to prevent undernutrition among young children (6 –23 months?**<br>□ Give more food □ Feed frequently □ Give attention during meals □ Other □ Don't know<br>□ Go to the health center/hospital and check that the child is growing (growth monitoring services)
**Attitudes towards a health or nutrition-related problem**
**Undernutrition - Perceived susceptibility**
**8. How likely do you think your child is to be undernourished, that is they stop growing or lose weight?**<br>□ 1. Not likely □ 2. You're not sure □ 3. Likely
**Undernutrition - Perceived severity**
**9. How serious do you think undernutrition is for a baby's health?**<br>□ 1. Not serious □ 2. You're not sure □ 3. Serious
**KAP: Iron-deficiency anemia**
**Knowledge**

1. Have you heard about iron-deficiency anemia?

□ Yes □ No □ Don’t know/no answer

If Yes: Can you tell me how you can recognize someone who has anemia?

□ Less energy/weakness □ Paleness / pallor □ Spoon nails / bent nails

□ More likely to become sick (less immunity to infections)	□ Other	□ Don’t know

2. What are the health risks for infants and young children of a lack of iron in the diet?

□ Delay of mental and physical development □ Other □ Don’t know

3. What are the health risks for pregnant women of a lack of iron in the diet?

□ Risk of dying during or after pregnancy □ Difficult delivery □ Other □ Don’t know

4. What causes anemia?

□ Lack of iron in the diet/eat too little, not much □ Heavy bleeding during menstruation □ Other

□ Sickness/infection (malaria, hookworm infection, other infection such as HIV/AIDS) □ Don’t know

5. How can anaemia be prevented?

□ Eat/feed iron-rich foods/having a diet rich in iron □ Take/give iron supplements if prescribed

□ Treat other causes of anemia (diseases and infections), seek health-care assistance □ Other □ Don’t know

□ Continue breastfeeding (for infants 6–23 months old) □ Eat / give vitamin-C-rich foods during or right after meals

6. Can you list examples of foods rich in iron?

Organ meat

□ Liver □ Kidney □ Heart

Flesh meat

□ Beef □ Lamb □ Goat □ Rabbit □ Chicken □ Duck

Fish and seafood

□ Fresh fish □ Dried fish □ Canned fish □ Prawns □ Shrimps □ Seafood

7. When taken during meals, certain foods help the body absorb and use iron. What are those foods?

□ Vitamin-C-rich foods, such as fresh citrus fruits (orange, lemons, etc.) □ Other □ Don’t know

8. Some beverages decrease iron absorption when taken with meals. Which ones?

□ Coffee □ Tea □ Other □ Don’t know

9. I would like to ask you about particular foods you may eat on their own or as part of a dish. Yesterday, during

the day and night, did you eat any of the following?

Organ meat

Liver □ Yes □ No **Kidney** □ Yes□ No **Heart**□ Yes □ No

Flesh meat

Beef □ Yes □ No **Lamb** □ Yes □ No **Goat**□ Yes □ No

Rabbit □ Yes □ No **Chicken** □ Yes □ No **Duck** □ Yes □ No

Fish and seafood

Fresh fish □ Yes □ No **Dried fish** □ Yes □ No **Canned fish** □ Yes□ No

Prawns □ Yes □ No **Shrimps** □ Yes □ No **Seafood** □ Yes □ No

10. Do you usually eat fresh citrus fruits, such as lemons, grapefruit, etc., or drink juice made from them?

□ Yes □ No □ Don't know / no answer

If Yes: Every day? □ Yes □ No □ Don't know / no answer

11. When do you usually eat fresh citrus fruits?

□ Before a meal □ During the meal □ After a meal □ Other □ Don't know/no answer

12. Do you usually drink coffee or tea?

□ Yes □ No □ Don't know

If Yes: Every day? □ Yes □ No □ Don't know

13. When do you usually drink coffee or tea?

□ Two hours or more before a meal □ Right before a meal □ During the meal

□ Right after a meal □ Two hours or more after a meal □ Other □ Don't know/no answer

Attitudes

Iron-deficiency anemia - Perceived susceptibility

14. How likely do you think your child is to be iron-deficient/anemic? OR How likely do you think you are to be iron-deficient / anemic?

□ 1. Not likely □ 2. You're not sure □ 3. Likely

If Not likely: Can you tell me the reason why it is not likely? ………………………………………………………...

Iron-deficiency anemia - Perceived severity

15. How serious do you think iron-deficiency/anemia is?

□ 1. Not serious □ 2. You're not sure □ 3. Serious

Attitudes towards an ideal or desired nutrition-related practice

Preparing meals with iron-rich foods - Perceived benefits

16. How good do you think it is to prepare meals with iron-rich foods such as beef, chicken or liver?

□ 1. Not good □ 2. You're not sure □ 3. Good

Preparing meals with iron-rich foods - Perceived barriers

17. How difficult is it for you to prepare meals with iron-rich foods?

□ 1. Not difficult □ 2. So-so □ 3. Difficult

Self-confidence

18. How confident do you feel in preparing meals with iron-rich foods?

□ 1. Not confident □ 2. Ok / so-so □ 3. Confident

Attitudes towards food preference

19. How much do you like the taste of [iron-rich food item or meal]?

□ 1. Dislike □ 2. You're not sure □ 3. Like

KAP: Vitamin A deficiency

Knowledge

1. Have you heard about vitamin A deficiency or lack of vitamin A?

□ Yes □ No □ Don't know / no answer

If Yes: Can you tell me how you can recognize someone who lacks vitamin A in his or her body?

□ Weakness / feels less energetic □ Be more likely to become sick (less immunity to infections)

□ Eye problems: night blindness (inability to see at dusk and in dim light), dry eyes, corneal damage, blindness

□ Other □ Don't know

2. What causes a lack of vitamin A in the body?

□ Poor variety of foods □ Eat too little food/not eat much (poor intake) □ Other □ Don't know

3. How can one prevent a lack of vitamin A in the body?

□ Eat / feed vitamin-A-rich foods – having / giving a diet rich in vitamin A □ Don't know

□ Eat / feed foods fortified with vitamin A □ Give vitamin A supplements/sprinkles □ Other

4. Do you know of any animal-source foods, vegetables or fruits that are rich in vitamin A?

Animal-source foods

□ Liver □ Kidney □ Heart

□ Milk, cheese, yogurt or other dairy product □ Egg yolks / egg from chicken, duck or other bird

Orange-coloured vegetables

□ Orange sweet potato □ Carrot □ Pumpkin □ Squash

Green vegetables

□ Spinach, kale and other green leafy vegetables

Fruits (orange- or yellow-coloured non-citrus fruits)

□ Ripe mango □ Ripe papaya □ Cantaloupe □ Apricot □ Dried peach

Foods fortified with vitamin A

□ Cereal grains □ Refined sugar and milk □ Edible oils and fats

Other foods

□ Breast milk (for infants 0–6 months) □ Other □ Don't know

Practices

5. Yesterday, during the day and night, did you eat any of the following foods?

Animal-source foods

Liver □ Yes □ No **Heart** □ Yes □ No **Milk, cheese, yogurt or other dairy product** □ Yes □ No

Egg yolks / egg from chicken, duck or other bird □ Yes □ No **Kidney** □ Yes □ No

Orange-coloured vegetables

**Orange sweet potato** □ Yes □ No **Carrot** □ Yes □ No<br><br>**Pumpkin** □ Yes □ No **Squash** □ Yes □ No<br><br>**<u>Green vegetables</u>**<br><br>**Spinach, kale and other green leafy vegetables** □ Yes□ No<br><br>**<u>Fruits (orange- or yellow-coloured non-citrus fruits)</u>**<br><br>**Ripe mango** □ Yes □ No **Ripe papaya** □ Yes □ No **Cantaloupe** □ Yes □ No<br><br>**Apricot** □ Yes □ No **Dried peach** □ Yes □ No<br><br>**<u>Foods fortified with vitamin A</u>**<br><br>**Cereal grains** □ Yes □ No **Refined sugar and milk** □ Yes □ No **Edible oils and fats** □ Yes □ No
**Attitudes**
**Vitamin A deficiency - Perceived susceptibility**
**6. How likely do you think your child is to lack vitamin A in his/her body? OR How likely do you think you are to lack vitamin A in your body?**<br><br>□ 1. Not likely □ 2. You're not sure □ 3. Likely
**Vitamin A deficiency - Perceived severity**
**7. How serious do you think a lack of vitamin A is?**<br><br>□ 1. Not serious □ 2. You're not sure □ 3. Serious
**Preparing meals with vitamin-A-rich foods - Perceived benefits**
**8. How good do you think it is to prepare meals with vitamin-A-rich foods such as carrots, green leafy vegetables, sweet-potatoes or liver?**<br><br>□ 1. Not good □ 2. You're not sure □ 3. Good
**Preparing meals with vitamin-A-rich foods - Perceived barriers**
**9. How difficult is it for you to prepare meals with vitamin-A-rich foods?**<br><br>□ 1. Not difficult □ 2. So-so □ 3. Difficult
**Self-confidence**

10. How confident do you feel in preparing meals with vitamin-A-rich foods?

□ 1. Not confident □ 2. Ok/so-so □ 3. Confident

Attitudes towards food preference

11. How much do you like the taste of [insert a vitamin-A-rich food item or meal]? Do you dislike it, youneither like it nor dislike it (neutral) or do you like it?

□ 1. Dislike □ 2. Neutral □ 3. Like

KAP: Food safety

Practices

1. After you have prepared dinner, kitchen surfaces, pots, pans, plates and utensils are dirty. Can you describe how you clean them usually?

□ Scrape excess food into rubbish bin □ Wash with hot water

□ Wash with detergent □ Don’t know/no answer

2. How do you store perishable fresh foods such as raw meat, poultry and seafood?

□ In the refrigerator (below 5 °C)/cool box □ Covered (protected from insects, rodents, pests and dust)

□ Separated from cooked or ready-to-eat foods □ Other □ Don’t know/no answer

Knowledge

3. Why should you prevent raw meat, offal, poultry and seafood from touching other foods such as those that are cooked or ready to eat?

□ Raw animal foods often contain germs (which may be transferred to cooked and ready-to-eat foods)

□ Other □ Don’t know

4. When cooking soups and stews, what sign shows that these are ready and safe to be served?

□ They are boiling / well-cooked □ Other □ Don’t know

5. What kinds of food should be placed in the refrigerator or in a cool place, such as an icebox or cool box?

Perishable foods

□ Meat □ Poultry □ Fish □ Foods from the sea or lake

□ Milk / dairy products	□ Cooked foods	□ Other	□ Don't know

6. Why should someone avoid eating leftovers that were not kept in a cool place?

□ Because food is not safe anymore □ Higher temperatures make germs grow faster

□ Foods get spoiled (germs multiply very quickly and can cause illness) □ Other □ Don't know

7. What should you do before eating raw fruits and vegetables?

□ Wash them with clean water □ Other □ Don't know

Attitudes

Food poisoning / sickness from eating spoiled food - Perceived susceptibility

8. How likely do you think you are to get sick from eating spoiled food?

□ 1. Not likely □ 2. You're not sure □ 3. Likely

Food poisoning / sickness from eating spoiled food - Perceived severity

9. How serious do you think it is to be sick from eating spoiled food?

□ 1. Not serious □ 2. You're not sure □ 3. Serious

Keeping perishable food in a cool place, for example in a cool box or in the refrigerator - Perceived benefits

10. How good do you think it is to keep meat, poultry, fish, seafood or cooked food in a cool place, for example in a cool box or in the refrigerator?

□ 1. Not good □ 2. You're not sure □ 3. Good

Keeping perishable food in a cool place, for example in a cool box or in the refrigerator - Perceived barriers

11. How difficult is it for you to keep these foods in a cool box or in the refrigerator?

□ 1. Not difficult □ 2. So-so □ 3. Difficult

Reheating leftovers before eating them - Perceived benefits

12. How good do you think it is to reheat leftovers before eating or serving them?

□ 1. Not good □ 2. You're not sure □ 3. Good

Reheating leftovers before eating them - Perceived barriers

13. How difficult is it for you to reheat leftovers before eating or serving them?

□ 1. Not difficult	□ 2. So-so	□ 3. Difficult
**Washing fruits and vegetables with clean water - Perceived benefits**		
**14. How good do you think it is to wash fruits and vegetables with clean water?**		
□ 1. Not good	□ 2. You're not sure	□ 3. Good
**Washing fruits and vegetables with clean water - Perceived barriers**		
**15. How difficult is it for you to wash fruits and vegetables with clean water?**		
□ 1. Not difficult	□ 2. So-so	□ 3. Difficult

First day: 24-hour dietary recall

Determine what was yesterday:

□ Saturday	□ Sunday	□ Monday	□ Tuesday	□ Wednesday	□ Thursday	□ Friday

Would you describe the food that you ate yesterday as atypical of your habitual food intake? □ Yes □ No

No.	Food Name	Time	Occasion (Breakfast – Launch – Dinner –Other)	Source of food (Homemade – Restaurant – Supermarket – Other)	Amount (According To Kitchen Staff)

Second day: 24-hour dietary recall

Determine what was yesterday:

□ Saturday	□ Sunday	□ Monday	□ Tuesday	□ Wednesday	□ Thursday	□ Friday

Would you describe the food that you ate yesterday as atypical of your habitual food intake? □ Yes □ No

No.	Food Name	Time	Occasion (Breakfast – Launch – Dinner –Other)	Source of food (Homemade – Restaurant – Supermarket – Other)	Amount (According To Kitchen Staff)
